# Supplementary material for: Enhanced critical current density in the pressure-induced magnetic state of the high-temperature superconductor FeSe
Source: Sci Rep. 2015 Nov 9;5:16385. doi: 10.1038/srep16385 (PMC4637863; doi:10.1038/srep16385)
Supplement: Supplementary Information [file srep16385-s1.pdf]

Supplementary Information for "Enhanced critical current density in the pressure-induced magnetic state of the high-temperature superconductor FeSe"

Soon-Gil Jung[1,*], Ji-Hoon Kang[1], Eunsung Park[1], Sangyun Lee[1], Jiunn-Yuan Lin[2], Dmitriy A. Chareev[3], Alexander N. Vasiliev[4,5,6], and Tuson Park[1,†]

[1]*Department of Physics, Sungkyunkwan University, Suwon 440-746, Republic of Korea*

[2]*Institute of Physics, National Chiao Tung University, Hsinchu 30010, Taiwan*

[3]*Institute of Experimental Mineralogy, Russian Academy of Sciences, Chernogolovka, Moscow Region 142432, Russia*

[4]*Low Temperature Physics and Superconductivity Department, Physics Faculty, Moscow State University, 119991 Moscow, Russia*

[5]*Theoretical Physics and Applied Mathematics Department, Institute of Physics and Technology, Ural Federal University, Ekaterinburg 620002, Russia*

[6]*National University of Science and Technology "MISiS", Moscow 119049, Russia*

*Corresponding author: Tuson Park (e-mail: tp8701@skku.edu), Soon-Gil Jung (e-mail: prosgjung@gmail.com)

In this supplement, we describe methods and show additional data and analyses that support the results in the main text.

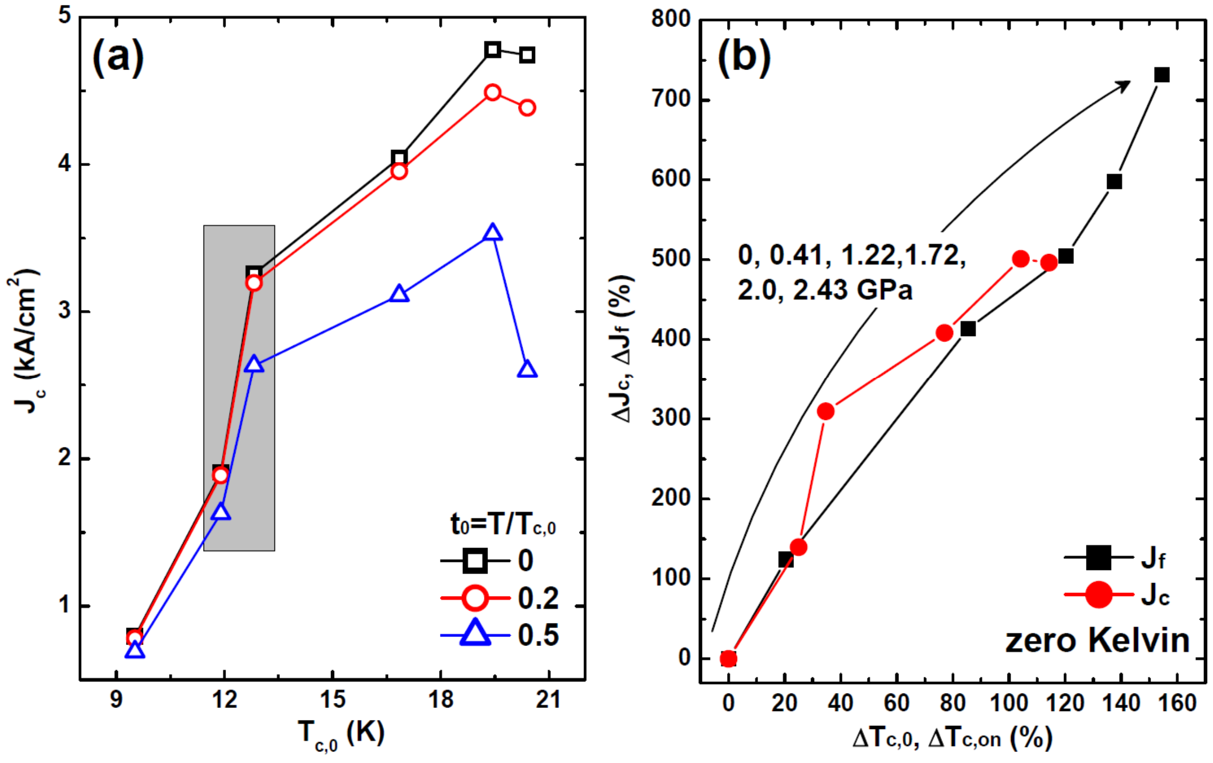

Figure S1. (a) The depinning critical current density J_c as a function of $T_{c,0}$ for reduced temperatures ($t_0=T/T_{c,0}$) of 0, 0.2 and 0.5. Here, $T_{c,0}$ is the zero-resistance temperature. Appearance of the sudden jump in J_c at 1.22 GPa, which is highlighted by the shade, is considered as due to magnetic pinning induced by the AFM phase. (b) Critical current densities of ΔJ_f and ΔJ_c as functions of $\Delta T_{c,on}$ and $\Delta T_{c,0}$, respectively, where $\Delta J_f = [J_f(P)\text{-}J_f(0)]/J_f(0)\times100\%$, $\Delta J_c = [J_c(P)\text{-}J_c(0)]/J_c(0)\times100\%$, $\Delta T_{c,on} = [T_{c,on}(P)\text{-}T_{c,on}(0)]/T_{c,on}(0)\times100\%]$, $\Delta T_{c,0} = [T_{c,0}(P)\text{-}T_{c,0}(0)]/T_{c,0}(0)\times100\%$, and $J_f(0)$, $J_c(0)$, $T_{c,on}(0)$, $T_{c,0}(0)$ are critical current densities and critical temperatures at ambient pressure. The J_f shows a monotonic increase with increase in $T_{c,on}$, on the other hand, J_c at 1.22 GPa shows a large enhancement despite a slight increase of $T_{c,0}$.

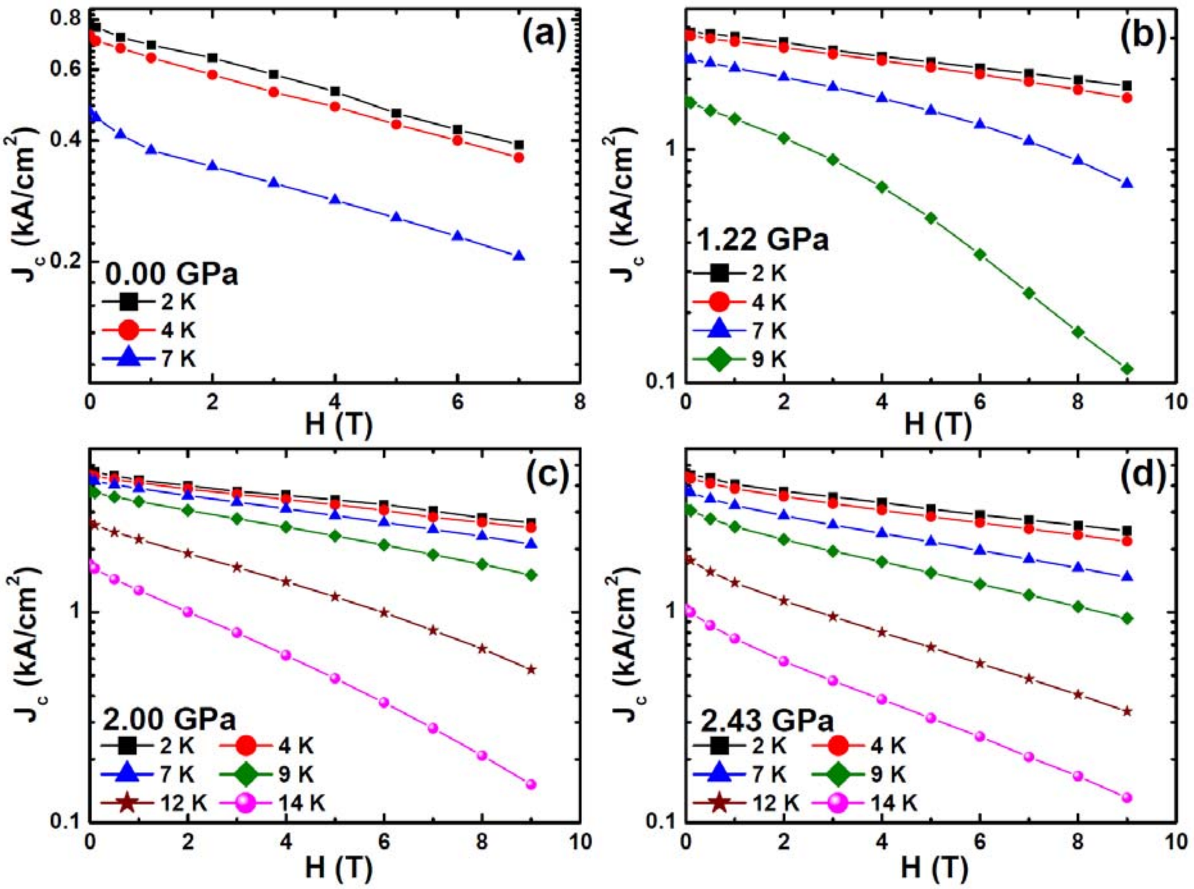

Figure S2. Depinning critical current density (J_c) of FeSe single crystals at pressures of (a) ambient, (b) 1.22 GPa, (c) 2.00 GPa, and (d) 2.43 GPa as a function of magnetic field. The weak-link behaviour that accompanies a sharp decrease in J_c at low fields is not observed over the entire pressure range. Magnetic fields were applied parallel to the *ab*-plane of FeSe.

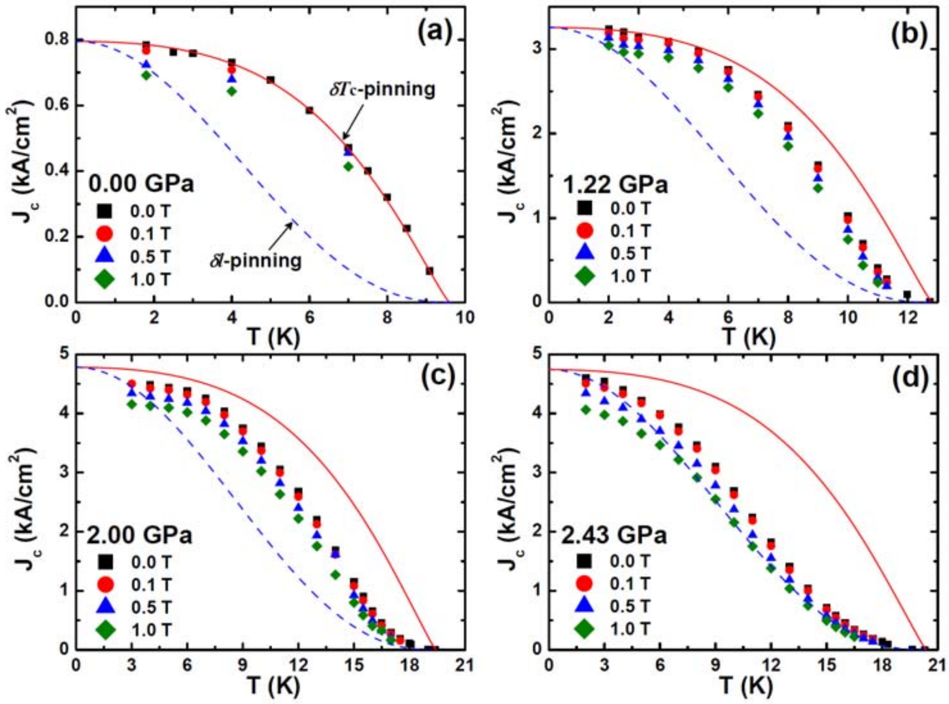

Figure S3. Temperature dependence of the depinning critical current density (J_c) under magnetic fields of 0 (squares), 0.1 (circles), 0.5 (triangles), and 1.0 Tesla (diamonds). The solid and the dashed lines are predictions from δT_c- and δl-pinning, respectively [S1,S2]. At ambient pressure, which is shown in panel (a), J_c is well explained by δT_c-pinning and its overall behaviour is almost independent of magnetic field, indicating that spatial fluctuations in T_c due to Se deficiencies and point defects are important to trapping vortices and that the pinning mechanism is insensitive to the field strength. When an antiferromagnetic phase is induced for pressures above 0.8 GPa [S3,S4], J_c can no longer be explained by δT_c-pinning, rather it follows δl-pinning, suggesting that magnetic interactions between vortices and Fe moments are important to the pinning mechanism. Panels (b), (c), and (d) show J_c at 1.22, 2.0, and 2.43 GPa, respectively. The two pinning mechanisms of δT_c-pinning and δl-pinning are related to the spatial fluctuations of the Ginzburg-Landau (GL) coefficient α associated with disorder in the superconducting transition temperature (T_c) and the spatial variations of the charge carrier mean free path (l) near lattice defects, respectively [S1,S2].

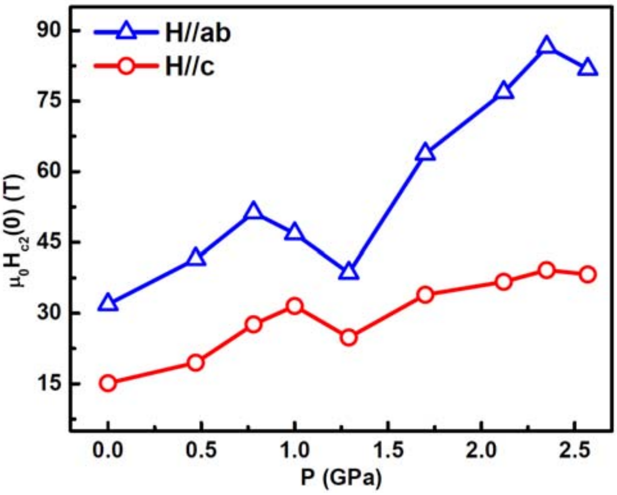

Figure S4. The zero temperature upper critical field, $\mu_0 H_{c2}(0)$, for FeSe as a function of applied pressure. $\mu_0 H_{c2}(0)$ is estimated from the Werthmaer Helfand and Hohenberg (WHH) model for magnetic field applied parallel (triangles) and perpendicular (circles) to the *ab*-plane [S5].

Supplementary references

[S1] Griessen, R. *et al*. Evidence for Mean Free Path Fluctuation Induced Pinning in $YBa_2Cu_3O_7$ and $YBa_2Cu_4O_8$ Films. *Phys. Rev. Lett*. **72**, 1910 (1994).

[S2] Blatter, G., Feigel'man, M. V., Geshkenbein, V. B., Larkin, A. I. & Vinokur, V. M. Vortices in High-temperature Superconductors. *Rev. Mod. Phys.* **66**, 1125 (1994).

[S3] Bendele, M. *et al*. Pressure Induced Static Magnetic Order in Superconducting $FeSe_{1-x}$. *Phys. Rev. Lett.* **104**, 087003 (2010).

[S4] Bendele, M. *et al*. Coexistence of Superconductivity and Magnetism in $FeSe_{1-x}$ under Pressure. *Phys. Rev. B* **85**, 064517 (2012).

[S5] Kang, J. -H. *et al*. (unpublished).
